# Risk factors for leukoplakia and malignant transformation to oral carcinoma: a leukoplakia cohort in Taiwan

**DOI:** 10.1054/bjoc.2000.1208

**Published:** 2000-06

**Authors:** M N Shiu, T H H Chen, S H Chang, L J Hahn

**Affiliations:** Graduate Institute of Epidemiology, College of Public Health, School of Dentistry, College of Medicine, National Taiwan University, Taipei, Taiwan

**Keywords:** leukoplakia, oral cancer, betel nut chewing, smoking

## Abstract

The effects of betel nut chewing, smoking and alcohol on the occurrence of leukoplakia and its malignant transformation to oral carcinoma were quantified in a leukoplakia cohort (*n* = 435) from one medical centre between 1988 and 1998 in Taiwan. Sixty oral carcinomas were ascertained in this cohort. A case–control study within the leukoplakia cohort was used to study, risk factors. Using the Weibull survival model, the incidence of malignant transformation of leukoplakia was shown to increase with follow-up years. After adjustment for other relevant risk factors, betel nut chewing (adjusted odds ratio (OR) = 4.59; 95% confidence interval (CI) 1.25–16.86) remained a significant risk factor for malignant transformation. Results from the case–control study showed that the adjusted odds ratios for betel nut chewing and smoking on the occurrence of leukoplakia were 17.43 (95% Cl 1.94–156.27) and 3.22 (95% Cl 1.06–9.78), respectively. Similar findings were observed when daily frequency and duration were taken into account. This implies that cessation of smoking may reduce by 36% leukoplakia cases, while elimination of betel nuts may prevent 62% of leukoplakia and 26% of malignant transformation to oral carcinoma in the underlying population. © 2000 Cancer Research Campaign


*British Journal of Cancer* (2000) 82(**11**), 1871–1874
© 2000 Cancer Research Campaign
DOI: 10.1054/ bjoc.2000.1208, available online at http://www.idealibrary.com on IDEAL®

### Risk factors for leukoplakia and malignant transformation to oral carcinoma: a leukoplakia cohort in Taiwan

**MN Shiu[1], THH Chen[1], SH Chang[1] and LJ Hahn[2]**

[1]Graduate Institute of Epidemiology, College of Public Health, and [2]School of Dentistry, College of Medicine, National Taiwan University, Taipei, Taiwan

**Summary** The effects of betel nut chewing, smoking and alcohol on the occurrence of leukoplakia and its malignant transformation to oral carcinoma were quantified in a leukoplakia cohort ($n = 435$) from one medical centre between 1988 and 1998 in Taiwan. Sixty oral carcinomas were ascertained in this cohort. A case–control study within the leukoplakia cohort was used to study, risk factors. Using the Weibull survival model, the incidence of malignant transformation of leukoplakia was shown to increase with follow-up years. After adjustment for other relevant risk factors, betel nut chewing (adjusted odds ratio (OR) = 4.59; 95% confidence interval (CI) 1.25–16.86) remained a significant risk factor for malignant transformation. Results from the case–control study showed that the adjusted odds ratios for betel nut chewing and smoking on the occurrence of leukoplakia were 17.43 (95% Cl 1.94–156.27) and 3.22 (95% Cl 1.06–9.78), respectively. Similar findings were observed when daily frequency and duration were taken into account. This implies that cessation of smoking may reduce by 36% leukoplakia cases, while elimination of betel nuts may prevent 62% of leukoplakia and 26% of malignant transformation to oral carcinoma in the underlying population. © 2000 Cancer Research Campaign

**Keywords**: leukoplakia; oral cancer; betel nut chewing; smoking

In Taiwan, the mortality from oral cancer has increased from 3.6 per 1000 in 1971 to 6.4 per 100 000 in 1994. Prevention of oral cancer seems imperative. It is not feasible to detect oral cancers early (Silverman, 1988; Shanta and Krishnamurthi, 1980) and mass screening for oral cancer has not been recommended (Warnakulasuriya and Johnson, 1996). Primary prevention via programmes to eliminate risk factors may become important (Warnakulasuriya and Johnson, 1996). Previous studies have demonstrated that smoking was highly associated with leukoplakia (Bouquot, 1987; Roed-Petersen, 1982; Brugere et al, 1986; Evstifeeva and Zaridze, 1992) but whether betel nut – containing arecoline, lime and piper (one kind of pepper) – was significantly related to leukoplakia has not been fully addressed. Since the consumption of betel nut in Taiwan has been increasing, elucidation of any association is becoming increasingly important. The association between alcohol consumption and leukoplakia is also inconclusive (Blot et al, 1988; Brugere et al, 1986; Evstifeeva and Zaridze, 1992).

Previous studies have shown a wide range of rates of malignant transformation of leukoplakia (0.13–36.4%) (Pindborg et al, 1968; Roed-Petersen, 1971; Banoczy, 1977; Gupta et al, 1980; Silverman et al, 1984; Bouquot et al, 1988). This suggests that the impact of putative risk factors may vary between different populations. The aims of this study were (1) to evaluate the effects of betel nut chewing, smoking and alcohol use on the risk of leukoplakia, taking the daily frequency and the duration of three factors into account; and (2) to assess the impact of relevant risk factors on malignant transformation to oral carcinoma, using samples from certain Chinese people in Taiwan.

*Received 1 September 1999*
*Revised 7 December 1999*
*Accepted 11 January 2000*

*Correspondence to:* THH Chen, Graduate Institute of Epidemiology, College of Public Health, National Taiwan University, Room 213, No 19 Hsuchow Road, Taipei, Taiwan

### MATERIALS AND METHODS

### Study design and subject selection

According to the WHO definition, a clinical diagnosis of leukoplakia is a keratotic white plaque that cannot be scraped off and cannot be given another specific diagnosis. Oral cancer is classified by the ICD into categories of lip, tongue, gum, mouth floor, buccal, palate, oropharynx, hypopharynx (International Code of Disease, ICD 140–149, excluding 142–147).

A total of 580 cases of leukoplakia diagnosed between June 1988 and February 1998 at one medical centre in Taiwan were ascertained. Of these, 145 patients who were subsequently diagnosed as having lichen planus, oral ulcer, infection, oral candidiasis or skin leukoplakia were excluded leaving 435 subjects in the leukoplakia cohort on which two parts of this study were based (Figure 1). Part A was to follow up the leukoplakia cohort to estimate the incidence of malignant transformation and relevant risk factors. Of the 435 patients, 60 oral carcinomas were identified, among which only 4% had a different location from the leukoplakia. In order to study factors accounting for malignant transformation, information was abstracted from the medical chart on date of diagnosis, location of leukoplakia and risk factors at time of leukoplakia diagnosis, such as betel nut chewing, smoking and alcohol use. These variables were routinely recorded in the medical chart.

Part B was to elucidate the association between risk factors and leukoplakia using a matched case–control study. A total of 100

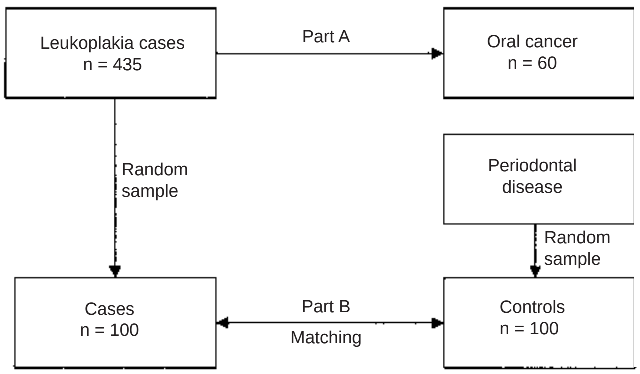

**Figure 1** The study design on factors affecting the risk leukoplakia and malignant transformation

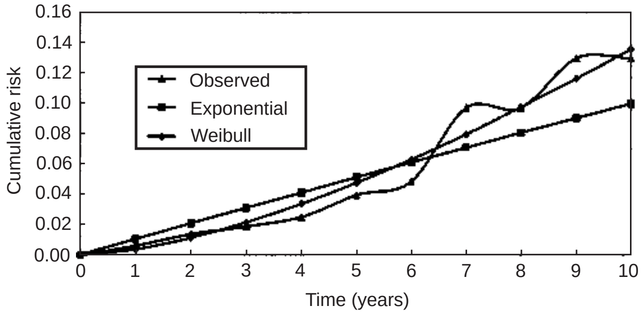

**Figure 2** Cumulative risk of malignant transformation, the observed and predicted using the exponential model and the Weibull Model

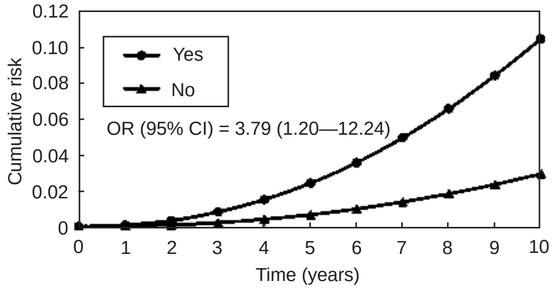

**Figure 3** Cumulative risk of malignant transformation by betel nut chewing leukoplakia cases were randomly selected from the above leukoplakia cohort. Each case was matched to one control for age (± 3 years), sex and date of diagnosis (± 3 months) derived from a total of 25 882 patients with a diagnosis of periodontal diseases in the same period and hospital as the study group. All cases and controls were free of oral cancer. Since information on risk factors such as betel nut chewing, smoking and drinking for the control group could not be completely obtained from medical charts we collected information via telephone interview. We also interviewed 68 out of 100 leukoplakia case–control pairs to check whether information from the medical chart was consistent with that from telephone interview. Reasons for non-participation included: not at home after three calls, wrong telephone number and refusal. Each participant was asked to provide information on the three risk factors, categorized as: current, former and never. Duration and daily frequency for each risk factor were also collected. A product of duration and daily frequency was defined as the intensity for the current and the former groups. The high or low intensity was categorized according to the median value for each risk factor. It should be noted that since there was a variety of types and brands for the drinkers, it was very difficult to define the dose per day. For alcohol use, only information on daily frequency was asked in telephone interview.

### Statistical methods

For Part A analysis, an accelerated failure time (AFT) model (Marubini and Valsecchi, 1995) was used to estimate the risk of malignant transformation and the association with the location of leukoplakia, betel nut chewing, smoking and drinking. Two parametric models including exponential and Weibull models were employed. All leukoplakia cases were followed until February 1998.

In the analysis of Part B, a conditional logistic regression was performed to investigate the relationship between betel nut chewing, smoking, alcohol drinking, and the occurrence of leukoplakia. The odds ratio (OR), together with exposure information derived from a previous population-based survey (Lee and Chen 1999), gave the population attributable proportion (PAR) for each risk factor (Breslow and Day 1980).

### RESULTS

### Malignant transformation from leukoplakia to oral carcinoma

Figure 2 shows that the incidence of malignant transformation in patients with leukoplakia increases with duration of follow-up.

The univariate analysis based on the Weibull model examined whether risk of malignant transformation depends on betel nut chewing, smoking, alcohol use and location of leukoplakia. The cumulative risk stratified by these variables was calculated. Figure 3 reveals that those who chewed betel nut were nearly four times more likely to develop malignant transformation than those who did not (OR = 3.79, 95% confidence interval (CI) 1.20–12.24). Other significant factors influencing malignant transformation include location around the tongue (OR = 3.65, 95% CI 1.09–12.25) and smoking (OR = 2.34, 95% CI 0.62–8.93).

Results from the multivariate analysis (Table 1), which incorporates significant factors in the above univariate analysis plus age and sex, show that betel nut chewing still remains a significant risk for malignant transformation. The hazard ratio for chewing betel nut was 4.59 (95% CI 1.25–16.85) after adjustment for age and sex. However, the effect of smoking and location on malignant transformation is not significant after adjustment for other factors.

### Risk factors for leukoplakia

Table 2 shows that the group of current betel nut chewers had a 26-fold higher (95% CI 3.27–204.00) risk for leukoplakia than that of the ‘never’ group. The OR for the occurrence of leukoplakia in

 *© 2000 Cancer Research Campaign*

**Table 1** Multivariate analysis of the impact of risk factors on the risk of malignant transformation in patients with leukoplakia, adjusted for age and sex using the Weibull regression model

| Risk factors | Odds ratio | 95% CI |
|---|---|---|
| Location | | |
| Tongue | 1.54 | 0.24–9.73 |
| Buccal | 0.30 | 0.05–1.72 |
| Alreola+lip | 0.57 | 0.06–5.83 |
| Others | 1 | |
| Betel nut chewing | | |
| Yes | 4.59 | 1.25–16.86 |
| No | 1 | |
| Cigarette smoking | | |
| Yes | 2.38 | 0.62–9.05 |
| No | 1 | |

the current smokers was 5.42 (95% CI 2.17–13.80). The frequent alcohol users had an ninefold (95% CI 1.11–68.7) risk for leukoplakia compared to 'never' group.

With respect to intensity, significant dose–response relationships were observed for three factors. For betel nut chewing, the risk for leukoplakia in the high intensity group (defined by a product of frequency and duration) was 32-fold (95% CI 2.44–408.00) higher than that in the 'never' group. The corresponding figure in the low intensity group was 16 (95% CI 1.37–137.00). As regards smoking, the high intensity group was five times more likely to develop leukoplakia than never smokers (95% CI 1.73–17.20), whereas the OR for the low intensity group compared with the never group was 3.68 (95% CI 1.20–11.20).

Table 3 presents the results of multivariate analysis after adjustment for the effect of the three variables on each other. The adjusted OR for chewing betel nut and smoking in the current group were 17.43 (95% CI 1.94–156.27) and 3.22 (95% CI 1.06–9.78) respectively. The adjusted OR for the use of betel nut and tobacco in the high intensity group were 22.49 (95% CI 1.44–351.42) and 3.09 (95% CI 0.93–10.34) respectively. The effect of alcohol on the occurrence of leukoplakia disappeared, after adjustment for betel nut chewing and smoking.

### Consistency of exposure information

Consistency of exposure information between the medical chart and telephone interview was checked. A good agreement was demonstrated by Kappa statistics (all Kappa values greater than 0.6). To establish whether two different procedures for obtaining information may have affected the results, we estimated the adjusted odds ratios based on the two sources and no significantly different results were found.

### DISCUSSION

A leukoplakia cohort study and the derived matched case–control were designed to elucidate the effect of betel nut chewing, smoking and alcohol use on the three-state natural history of oral cancer, form normal, through leukoplakia and to oral carcinoma. Results from this study had two major practical findings. First, chewing betel nut was demonstrated to influence not only the occurrence of leukoplakia but also its malignant transformation. Although smoking might play a major role in the occurrence of

**Table 2** Univariate analysis of the effect of cigarette smoking, betel nut chewing and alcohol use on the incidence of leukoplakia

| Risk factyor | | Odds ratio (95% CI) | | | |
|---|---|---|---|---|---|
| | | Status classification | | Intensity classification | |
| | Never | Former | Current | Low | High |
| Cigarette smoking | 1 | 2.03<br>(0.61–6.75) | 5.42<br>(2.17–13.80) | 3.68<br>(1.20–11.20) | 5.45<br>(1.73–17.20) |
| Betel nut chewing | 1 | 3.78<br>(0.61–23.30) | 25.85<br>(3.27–204.00) | 15.61<br>(1.77–137.00) | 31.55<br>(2.44–408.00) |
| | Never | Occasional | Frequent | | |
| Alcohol use | 1 | 0.63<br>(0.11–3.65) | 8.66<br>(1.11–68.70) | | |

**Table 3** Multivariate analysis of the effect of alcohol use, betel nut chewing and cigarette smoking on the incidence of leukoplakia, adjusted for the effects of three factors on each other

| Risk factor | | Odds ratio (95% CI) | | | |
|---|---|---|---|---|---|
| | | Status classification | | Intensity classification | |
| | Never | Former | Current | Low | High |
| Cigarette smoking | 1 | 1.04<br>(0.24–4.59) | 3.22<br>(1.06–9.78) | 1.67<br>(0.45–6.26) | 3.09<br>(0.93–10.30) |
| Betel nuts chewing | 1 | 2.38<br>(0.34–16.75) | 17.43<br>(1.94–156.27) | 9.06<br>(1.00–81.64) | 22.49<br>(1.44–351.00) |
| | Never | Occasional | Frequent | | |
| Alcohol use | 1 | 0.28<br>(0.03–2.56) | 3.00<br>(0.27–33.50) | | |

*© 2000 Cancer Research Campaign*

leukoplakia, it may not be a main contributory cause for malignant transformation. If the prevalence of smoking and betel nut chewing among the general population are estimated as 26% and 10% (Lee and Chen, 1999), respectively, this information plus ORs reveals that eliminating the habit of betel nut chewing may reduce the occurrence of leukoplakia by 62% and reduce the rate of malignant transformation by 26% in the underlying population. The corresponding figures for the elimination of smoking were 36% and 26% respectively. Thus, a primary prevention program designed to discourage people from betel nut chewing and smoking seems crucial.

Second, as the likelihood ratio test between the exponential model (constant hazard) and the Weibull model (increasing hazard) was statistically significant ($\chi^2_{(1)}$ = 4.30, $P$ = 0.038) (Figure 2) this implies that the incidence of malignant transformation from leukoplakia increases with time. Increased risk associated with increasing duration after diagnosis was most marked for leukoplakia in subjects with the habit of betel nut chewing. This might reflect the fact that the proportion of leukoplakia among individuals who chew betel nut also increases year by year.

Few studies have reported on the association between betel nut chewing and leukoplakia or the effect of betel nut chewing on malignant transformation. Only Silverman et al (1984) reported a statistically significant relationship between Pan (a mixture of tobacco and betel nut) and malignant transformation. However, as chewing betel nut in our study did not involve a mixture of tobacco, it is difficult to compare our results with Silverman et al.

A significant positive association between smoking and leukoplakia is consistent with previous reports (Evstifeeva and Zaridze, 1992; Kulasegaram et al, 1995). Our finding of a dose–response relationship is also in agreement with the study by Kulasegaram et al (1995).

The increased risk of malignant transformation with time in this study is at odds with the previous report that this decreased with time (Silverman et al, 1984). Three possibilities may be relevant. First, the histological distribution of leukoplakia in this study may be different. A persistence of betel nut chewing in leukoplakia cases in Taiwan may provide a second explanation. Third, different treatments might also affect the rate of transformation.

With respect to malignant transformation, only 4% of our leukoplakia cases did not have the same location as oral carcinoma. Such cancers may originate from other sites of leukoplakia but their small number is unlikely to have affected the results.

In conclusion, this study finds that betel nut chewing is a major risk factor not only for the occurrence of leukoplakia but also for malignant transformation. We estimate that elimination of betel nut chewing would prevent 62% of leukoplakia and 26% of malignant transformation.

### REFERENCES

Banoczy J (1977) Follow-up studies in oral leukoplakia. *J Maxillofac Surg* **5**: 69–75

Blot WJ, McLaughlin JK, Winn DM, et al (1988) Smoking and drinking in relation to oral and pharyngeal cancer. *Cancer Res* **48**: 3282–3287

Bouquot JE (1987) Epidemiology. In: *Pathology of the Head and Neck*, Gnepp DG (ed), Churchill Livingstone: New York; pp. 263–314

Bouquot JE, Weiland LH and Kurland LT (1988) Leukoplakia and carcinoma in situ synchronously associated with invasive oral/oropharyngeal carcinoma in Rochester, Minn, 1935–1984. *Oral Surg Oral Med Oral Path* **65**: 199–207

Breslow NE and Day NE (1980) Statistical methods in cancer research. volume 1 The analysis of case–control studies. International Agency for Research on Cancer, Lyon; p. 74

Brugere J, Guenel P, Leclerc A and Rodriguez J (1986) Differential effects of tobacco and alcohol in cancer of the larynx, pharynx and mouth. *Cancer* **57**: 391–395

Evstifeeva TV and Zaridze DG (1992) Nass use, cigarette smoking, alcohol consumption and risk of oral and oesophageal precancer. *Eur J Cancer* **28B**: 29–35

Gupta PC, Mehta FS, Daftary DK et al (1980) Incidence rates of oral cancer and natural history of oral precancerous lesions in a 10-year follow-up study of Indian villagers. *Community Dent Oral Epidemiol* **8**: 283–333

Kulasegaram R, Downer MC, Jullien JA, Zakrzewska JM and Speight PM (1995) Case–control study of oral dysplasia and risk habits among patients of a dental hospital. *Eur J Cancer* **31B**: 227–231

Lee L and Chen HH (1999) A survey of smoking and betel nut chewing in Taiwan. Technique report of the bureau of health promotion and protection DOH(DOH88-HP-Tobac03)

Marubini E and Valsecchi MG (1995) *Analysing Survival Data from Clinical Trials and Observational Studies*, pp: 267–292 John Wiley: London

Pindborg JJ, Jolst O, Renstrup G and Roed-Petersen B (1968) Studies in oral leukoplakia: a preliminary report on the period prevalence of malignant transformation in leukoplakia based on a follow-up study of 248 patients. *J Am Dent Asso* **76**: 767–771

Roed-Petersen B (1971) Cancer development in oral leukoplakia: Follow-up of 331 patients. *J Dent Res* **50**: 711

Roed-Petersen B (1982) Effect on oral leukoplakia of reducing or ceasing tobacco smoking. *Acta Dermato-Venereol* **62**: 164–167

Shanta V and Krishnamurthi S (1980) Combined bleomycin and radiotherapy in oral cancer. *Clinical Radiology* **31**: 617–620

Silverman SJ (1988) Early diagnosis of oral cancer. *Cancer* **62**: 1796–1799

Silverman SJ, Gorsky M and Lozada F (1984) Oral leukoplakia and malignant transformation: A follow-up study of 257 patients. *Cancer* **53**: 563–568

Warnakulasuriya KA and Johnson NW (1996) Strengths and weaknesses of screening programmes for oral malignancies and potentially malignant lesions. *Eur J Cancer Prev* **5**: 93–98

WHO Collaborating Centre for Oral Precancerous Lesions (1978) Definition of leukoplakia and related lesions: an aid to studies in oral precancer. *Oral Surg, Oral Medicine, Oral Pathol* **46**: 518–539

*© 2000 Cancer Research Campaign*
